# Digital Health Communication Engagement Experiences of Older Adults in Community Contexts: Qualitative Systematic Review and Meta-Ethnography

**DOI:** 10.2196/89179

**Published:** 2026-08-05

**Authors:** Xinxin Wang, Jianying Zhou, Kari Aerzuguli, Yufei Xing, Wei Luan, Qiong Fang

**Affiliations:** 1 Shanghai Jiao Tong University School of Nursing, No. 100, Tianxiong Road, Pudong New Area, Shanghai, 201318, China, 86 021-63846590; 2 Songjiang Hospital Affiliated to Shanghai Jiao Tong University School of Medicine, Shanghai, China; 3 Shuguang Hospital Affiliated to Shanghai University of Traditional Chinese Medicine, Shanghai, China

**Keywords:** health communication, older adults, qualitative research, systematic review, meta-ethnography, Enhancing Transparency in Reporting the Synthesis of Qualitative Research, ENTREQ

## Abstract

**Background:**

The global population is aging rapidly, straining health care and social systems. Amid digital transformation, older adults face pronounced obstacles to participating in and benefiting from health communication. Prior syntheses emphasized technology adoption or clinical effectiveness, and health communication reviews focused on formal or home-based care. How older adults experience digital health communication as an everyday, relational process in community contexts and how trust and responsibility take shape remain underexplored.

**Objective:**

This study aimed to synthesize the experiences of older adults engaging in digital health communication in community contexts.

**Methods:**

We searched PubMed, CINAHL, Embase, PsycINFO, Scopus, ProQuest Health & Medical Collection, Web of Science Core Collection, CNKI, and Wanfang from inception to June 2026. Qualitative and mixed methods studies on the digital health communication experiences of community-dwelling older adults (aged ≥50 y) were included; purely quantitative studies were excluded. Two researchers independently screened records, appraised the studies using the CASP (Critical Appraisal Skills Programme) tool, and extracted qualitative data. Findings were synthesized using the Noblit and Hare meta-ethnography, and confidence was assessed using the GRADE-CERQual (Grading of Recommendations Assessment, Development, and Evaluation—Confidence in the Evidence from Reviews of Qualitative Research) approach. The review was registered with PROSPERO and reported following PRISMA (Preferred Reporting Items for Systematic Reviews and Meta-Analyses), PRISMA-S (PRISMA Extension for Reporting Literature Searches in Systematic Reviews), and ENTREQ (Enhancing Transparency in Reporting the Synthesis of Qualitative Research).

**Results:**

The integration of 14 studies across 8 countries yielded 4 themes. Older adults’ objectives in community digital health communication encompass obtaining or sharing health information, maintaining health, and learning technology to avoid falling behind. Engagement enhanced health management, improved access to health care services, knowledge, and skills, and increased social participation. Trust was built primarily on patient-provider relationships and authoritative platforms, while accountability was distributed across individuals, families, health care providers, and communities, despite imbalances such as technological dependency and difficulty verifying information. Adaptation to technology was dual in nature: social support, patient-provider trust, and technological affinity were key drivers, whereas insufficient digital literacy, technology anxiety, cost constraints, physiological limitations, and privacy concerns constituted significant barriers.

**Conclusions:**

This meta-ethnography treats community-based digital health communication as a communicative practice in its own right. It reframes the community not as a setting in which communication occurs but as a determinant of whether it works and characterizes engagement as an evolving negotiation of motivation, trust, and responsibility. Findings argue for moving beyond efficiency-oriented service delivery toward building community capacity and supporting older adults’ autonomy and digital health literacy. Twelve out of 14 studies were of moderate quality, and eligibility was limited to Chinese- or English-language abstracts, which may limit representativeness.

## Introduction

With the significant increase in global life expectancy, the continuous expansion of the older population has become an undeniable social phenomenon. According to the United Nations’ World Population Prospects 2024, the number of people aged 65 years and older worldwide is projected to reach 2.2 billion by the late 2070s, surpassing the number of children below the age of 18 years [1]. This profound demographic shift poses sustained pressure on health care systems and creates an urgent need for scalable strategies that strengthen older adults’ health literacy and access to health information [2].

Health communication is one of the most cost-effective public health strategies for translating health knowledge, promoting behavioral change, and enhancing health literacy [3-5]. For older adults specifically, health communication not only facilitates their reception, understanding, and application of personalized health information, but also influences their attitudes and cognition, thereby promoting health behavior change and achieving health promotion outcomes [6]. The rapid advancement of digital technologies, including AI, virtual and augmented reality, and machine learning, has transformed how health communication is practiced [7]. Digital technology mediates and embeds itself within everyday social life and human communicative behavior, reshaping individuals’ experiences of time and space, as well as physical and mental dimensions, and subject-object relationships through technological participation [8], creating a dynamic, multidimensional ecosystem through which health information now circulates [9]. However, older adults face a wider digital gap than younger groups, along with intergenerational communication barriers and information-cocoon effects, which can deepen their isolation and widen health inequalities [7,10-12]. In this review, “older adults” refers to people aged 50 years or older. This threshold follows the operational definition adopted by the World Health Organization’s (WHO)’s Study on Global Ageing and Adult Health (SAGE) [13]. It is also consistent with recent qualitative reviews on digital aging that adopt this threshold to capture the wide range of experiences from the early-old to the oldest-old [14].

Digital health communication refers to the use of digital technologies, such as mobile health apps, telehealth platforms, patient portals, social media, and online health communities, to support the exchange, dissemination, and acquisition of health information among older adults, health care providers, and peers [5,15]. This focus is distinct from the category of digital health interventions that actively manage clinical or behavioral outcomes, such as remote monitoring, digital therapeutics, and AI-based clinical decision support [16,17]. Existing qualitative syntheses have offered valuable insights into this field, yet have largely approached it from particular angles. Much of this work has centered on the determinants of adoption or on effectiveness: Aslan et al [18] used meta-ethnography to identify the barriers and facilitators of older adults’ adoption of communicative e-health services in clinical encounters, and reviews of digital health technologies similarly emphasized effectiveness within clinical care pathways [19,20]. Other reviews have examined health communication within specific care or interaction settings, such as home health care services [21] and primary care settings [22]. Across this body of work, 3 issues remain underexamined. First, the community has been treated mainly as a backdrop, rather than as the everyday setting in which older adults access, share, and make sense of health information. Second, older adults’ experiences of trust and responsibility have been examined chiefly at the point of technology adoption, rather than across the fuller communicative process that follows. Third, the communicative use of digital technology has rarely been disentangled from its use as a clinical or behavioral intervention. As digital health platforms are now embedded in routine community life [19] and continue to reshape how older adults reach health information [23], an updated qualitative synthesis focused specifically on community-based digital health communication is warranted.

This systematic review and meta-ethnography aim to synthesize and interpret existing qualitative evidence to understand the experiences of older adults participating in community-based digital health communication and to generate transferable conceptual insights to inform age-friendly digital health services and policy.

## Methods

### Study Design

This systematic review was conducted and reported in accordance with the PRISMA (Preferred Reporting Items for Systematic Reviews and Meta-Analyses) 2020 statement (Checklist 1) and its expanded checklist (Checklist 2) [24], the PRISMA 2020 for Abstracts checklist (Checklist 3) [24], the PRISMA-S (PRISMA Extension for Reporting Literature Searches in Systematic Reviews; Checklist 4) [25], and the ENTREQ (Enhancing Transparency in Reporting the Synthesis of Qualitative Research; Checklist 5) statement [26]. Because this review employed a qualitative synthesis approach without meta-analysis, the SWiM (Synthesis Without Meta-Analysis; Checklist 6) reporting guideline was also followed [27]. This review deviated from the protocol registered in PROSPERO (registration number CRD420251002601) in 2 respects. First, the age criterion for participant inclusion was broadened from “60 years or older” to “50 years or older” during screening to capture the heterogeneous experiences spanning the early-old to the oldest-old. Second, 2 additional databases (PsycINFO and Scopus) were added to the original search strategy to enhance the comprehensiveness of the literature retrieval. These amendments were implemented during the search and screening stages of the review and did not alter the overall research questions or synthesis approach.

### Eligibility Criteria

Inclusion criteria for the study were (1) research topic: exploring older people’s experiences participating in community digital health communication; (2) participants were older adults (aged ≥50 y) residing in the community for over 6 months or members of a subgroup, regardless of health status, socioeconomic background, or digital technology experience; (3) qualitative or mixed methods research designs were used, and relevant qualitative data could be extracted. Exclusion criteria were (1) purely quantitative studies, case reports, and reviews; (2) studies where full-text access was unavailable or data were incomplete. If a study evaluated a broader or multicomponent program (eg, a comprehensive health education intervention) that combined communicative and intervention elements, it was not excluded on that basis; in such cases, only the participant accounts concerning digital health communication were extracted and synthesized at the data-extraction stage.

### Information Sources

Systematic searches were conducted in PubMed (NLM), CINAHL (EBSCOhost), Embase (Elsevier), PsycINFO (EBSCOhost), Scopus (Elsevier), ProQuest Health & Medical Collection (ProQuest), Web of Science Core Collection databases (Clarivate), CNKI (China National Knowledge Infrastructure), and Wanfang Data. No study registries were searched. No additional studies or data were sought by contacting authors, experts, or manufacturers. The literature search period spanned from the establishment of each database to June 2026. Researchers manually searched the references, research protocols, and gray literature of relevant systematic reviews. Screening records that cited the included studies was also performed. Each database was searched separately, and no databases were searched simultaneously on a shared platform.

### Search Strategy

A combination of subject headings and expanded terms was employed, including search terms such as “AI, digital, e-health, online, health knowledge, medical advice, wellness facts, popular*, disseminat*, public participation,” among others. The search strategy was partly informed by a previous meta-synthesis of qualitative research [28], but we did not use published search filters. The full search strategies for each database are provided in Multimedia Appendix 1. Studies with abstracts published in Chinese or English were searched due to the language competencies of the review team. The initial search was conducted in March 2025. The search was rerun in May 2026 and June 2026 to capture any new studies published since the initial search. No other date or study-design restrictions were applied. The search strategy was not formally peer-reviewed using the PRESS (Peer Review of Electronic Search Strategies) checklist. However, it was iteratively refined through internal team discussions involving a research librarian and a senior researcher experienced in systematic reviews.

### Selection Process

EndNote X9 (Clarivate Analytics) software was used for deduplication. Two researchers independently screened study titles, abstracts, and full texts of eligible studies. In cases of disagreement, a third researcher made the final decision.

### Data Collection Process

Two researchers independently conducted detailed readings of the included studies, identifying their metaphors, concepts, and themes. Content extraction was performed using Microsoft Excel 2019. Disputes were resolved through discussions between the 2 researchers. No automation tools were used in the data collection process. The template data extraction form is provided in Multimedia Appendix 2.

### Data Items

The following study-level characteristics were extracted from each included study: first author, year of publication, country or region, study aim, study design, participant characteristics (sample size, age range, gender composition, health status, and digital technology experience), data collection method, analytical approach, and key qualitative findings (Multimedia Appendix 3).

As this is a qualitative meta-ethnography, no prespecified quantitative outcome measures or effect sizes were sought. The qualitative data items comprised 2 levels: first-order constructs, defined as direct quotations from study participants reflecting their lived experiences; and second-order constructs, defined as the interpretive themes and concepts developed by the original study authors based on participant data [29,30]. For studies that evaluated broader or multicomponent programs, only data concerning participants’ experiences of digital health communication—that is, their reception, exchange, understanding, and the use of health information—were extracted. Data relating exclusively to the noncommunicative components of an intervention were not extracted or synthesized. Where information was missing or unclear in the original reports, this was recorded in the data extraction form.

### Study Risk of Bias Assessment

The quality of the included studies was assessed using the CASP (Critical Appraisal Skills Programme) qualitative research checklist (Multimedia Appendix 4) [31]. Two researchers independently assessed the quality of each included study, with disagreements resolved through discussion. All studies were incorporated into the data synthesis to enhance the depth and breadth of the meta-ethnography synthesis regardless of their quality scores [30].

### Effect Measures

This review employed a qualitative meta-ethnographic synthesis approach rather than a quantitative meta-analysis. Accordingly, the present synthesis does not report effect measures in the conventional sense; the “effects” reported here take the form of interpretive themes and higher-order conceptual constructs derived from the synthesis of qualitative findings across the included studies, rather than quantifiable effect estimates.

### Synthesis Methods

Our study adopted the Noblit and Hare [29] meta-ethnography as a qualitative synthesis method. Meta-ethnography is an interpretive approach to synthesizing qualitative research, originally developed by Noblit and Hare in 1988. Unlike aggregative approaches that merely summarize findings, meta-ethnography seeks to translate studies into one another by identifying and comparing key concepts, metaphors, and themes across studies, thereby generating new interpretive understandings that go beyond the findings of individual studies [29]. This inductive and interpretive approach allows for the development of conceptual understanding [30] and has been extensively used in synthesizing qualitative health care research [32]. The eMERGe (Meta-Ethnography Reporting Guidance) framework, which provides a set of structured reporting criteria specifically designed to improve the completeness and clarity of meta-ethnography reports, was used to guide the reporting of this review [33].

The data synthesis followed the phases of meta-ethnography: reciprocal translation, refutation, and line-of-argument syntheses [29]. Two researchers independently and repeatedly reviewed each included study and extracted its first-order and second-order constructs. Through line-by-line comparison across studies, conceptually similar constructs were grouped. Subsequently, using a cross-textual comparative approach, the researchers identified recurring concepts and metaphors while examining similarities and differences in research contexts to establish logical connections between themes and build third-order interpretations. Discrepancies at every stage were resolved through discussion, and the developing themes were checked against the original study data.

### Reporting Bias Assessment

To mitigate potential reporting bias, we comprehensively searched 9 electronic databases and supplemented this with manual reference searching and gray literature screening. The risk of selective nonreporting of qualitative findings within individual studies was addressed as part of the CASP quality appraisal and the GRADE-CERQual (Grading of Recommendations Assessment, Development, and Evaluation—Confidence in the Evidence from Reviews of Qualitative Research) adequacy of data assessment.

### Certainty Assessment

We employed the GRADE-CERQual framework to assess the confidence in evidence [34]. Two researchers independently assessed the confidence in evidence for each review finding. The GRADE-CERQual assessment considered: (1) methodological limitations, (2) relevance, (3) coherence, and (4) adequacy of data. Based on these 4 components, an overall confidence assessment was made for each finding, categorized as high, moderate, low, or very low confidence. Each finding started with high confidence and was downgraded according to concerns identified across the 4 components, with the final rating agreed upon through discussion.

## Results

### Study Selection

**Figure 1.**
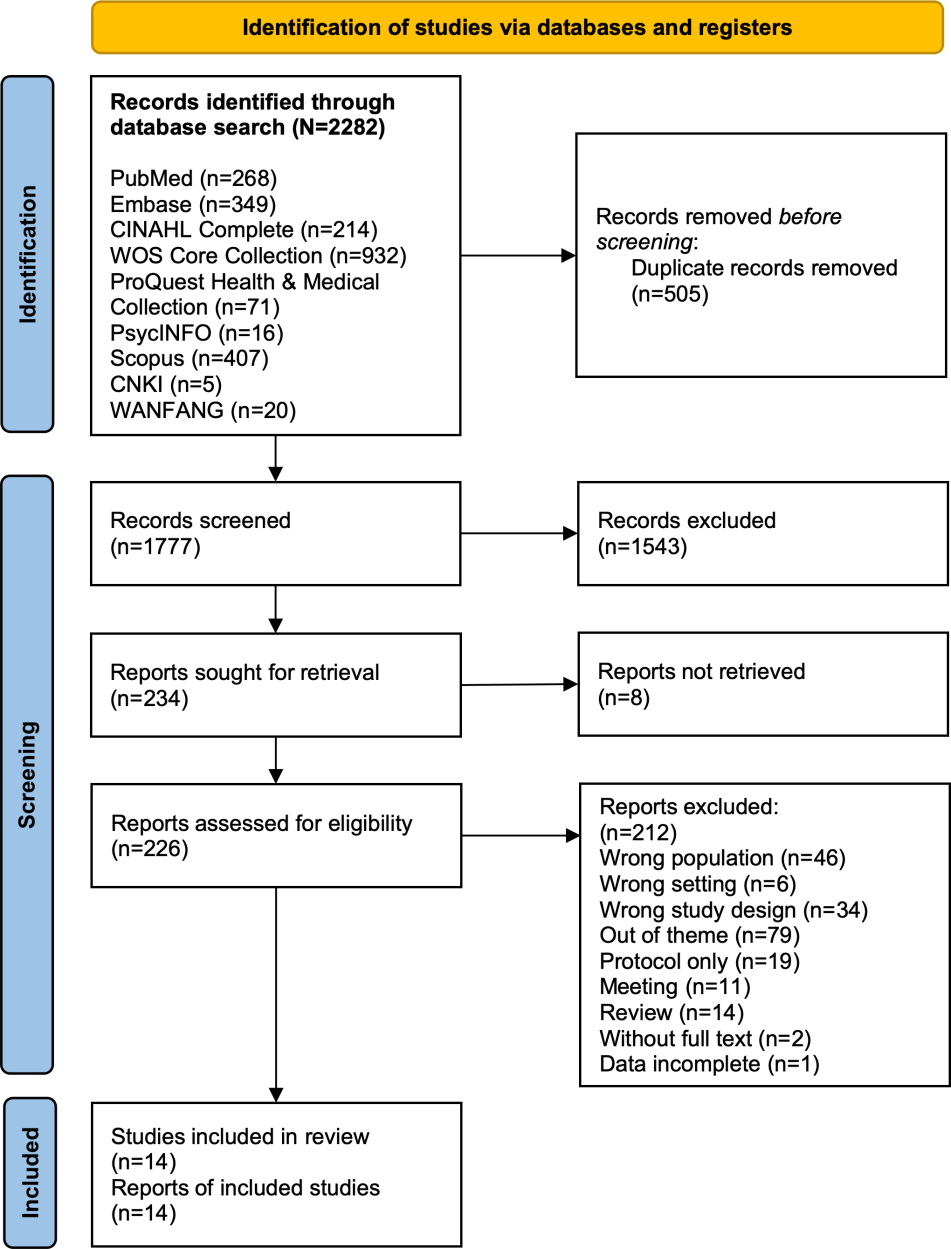
PRISMA (Preferred Reporting Items for Systematic Reviews and Meta-Analyses) flow diagram of study selection for the qualitative systematic review.

This systematic review utilized the PRISMA flow diagram to illustrate the literature screening process (Figure 1). The initial search yielded 2282 records. After removing duplicates, 1777 studies were selected for title and abstract screening. Ultimately, 14 studies meeting the inclusion criteria were incorporated into this systematic review.

### Study Characteristics

All included studies incorporated qualitative research designs, with 3 using mixed methods approaches. Seven studies used thematic analysis, 3 applied content analysis, 1 adopted inductive analysis, 1 adopted deductive analysis, 1 adopted codebook thematic analysis, and another followed grounded theory methodology. The majority of the research was conducted in North America (n=7), followed by Asia (n=5) and Europe (n=2; Multimedia Appendix 3).

### Quality Assessment of Included Studies

The average CASP score for studies included in this systematic review was 17.7 (SD 1.3) points, ranging from 15 to 20 points. Twelve studies were rated as moderate quality, while only 2 were classified as high quality (total scores of 19 or 20; Multimedia Appendix 4). Among these, 8 studies did not adequately address the relationship between researchers and participants, specifically, the researchers’ roles, potential biases, and influences during subject selection; how they managed events occurring during the study period; and whether they considered the impact of any changes to the study design.

### Confidence in the Review Findings

Table 1 presents the confidence assessment of research findings based on the GRADE-CERQual methodology. As shown in Table 1, the overall confidence in the evidence was rated as high for all 4 themes, with all included studies demonstrating methodological quality at moderate or higher levels. Furthermore, the included studies feature rich interview data from diverse countries, alleviating concerns regarding adequacy.

**Table 1.** GRADE-CERQual (Grading of Recommendations Assessment, Development, and Evaluation—Confidence in the Evidence from Reviews of Qualitative Research) confidence level of the review findings.

Review finding (number of articles, n)	Confidence level	Explanation of GRADE-CERQual assessment	Including studies
Core objectives (n=9)	High confidence	Nine studies with moderate-to-high methodological quality Thick data from 6 countries High coherence and truly reflect the review question	[35-43]
Behavioral outcomes (n=11)	High confidence	Eleven studies with moderate-to-high methodological quality Thick data from 6 countries High coherence and truly reflect the review question	[35-38,40-46]
Trust and responsibility (n=13)	High confidence	Thirteen studies with moderate-to-high methodological quality Thick data from 8 countries High coherence and truly reflect the review question	[35-47]
Adapting to digital technology (n=14)	High confidence	Fourteen studies with moderate-to-high methodological quality Thick data from 8 countries High coherence and truly reflect the review question	[35-48]

### Results of Meta-Ethnographic Synthesis

Through the systematic integration of the included studies, 4 core themes were identified: core objectives, behavioral outcomes, trust and responsibility, and adapting to digital technologies. These themes comprehensively reveal the motivations, practical behavioral impacts, trust relationship dynamics, and the facilitating and hindering forces in the adoption of digital health technologies among community-dwelling older adults. Following the Noblit and Hare [29] synthesis process, the analysis revealed that the relationships across studies were predominantly reciprocal rather than refutational. No direct contradictions between studies were identified; instead, the included studies consistently reinforced and extended one another’s findings within each theme. Specifically, the unifying concept of “core objectives” centers on older adults’ proactive pursuit of health information and technological competence; “behavioral outcomes” converges on multidimensional empowerment spanning health management, social participation, and skill acquisition; “trust and responsibility” is unified by the interplay between authority-based trust and multistakeholder accountability; and “adapting to digital technology” shares a common dual-factor structure of promoting and barrier forces. Table 2 illustrates the cross-study construction process for each theme.

**Table 2.** Meta-ethnographic synthesis of findings across 14 included studies.

Included studies	Zibrik et al [47] (2015), Canada	Zhang et al [35] (2023), Singapore	Pan et al [39] (2024), Korea	Neumann et al [40] (2023), America	Glowackie et al [45] (2021), America	Cheng et al [42] (2023), Hong Kong, China	Bernhard et al [43] (2017), Germany	Arthanat et al [48] (2024), America	Akenine et al [46] (2020), Europe (Finland, France, and Netherlands)	Saravanan et al [44] (2024), America	Kyaw et al [41] (2026), Singapore	Philip et al [38] (2025), Canada	Wang et al [37] (2025), China	Yang et al [36] (2026), Canada
Theme 1: core objectives														
Obtain or share health information		✓	✓	✓		✓	✓				✓	✓	✓	
Maintain health				✓			✓				✓		✓	
Learn technology to avoid falling behind			✓	✓		✓								✓
Theme 2: behavioral outcomes														
Access to medical resources and services		✓		✓		✓	✓				✓			✓
Improve health status				✓			✓				✓		✓	✓
Promotion of self-management				✓			✓		✓	✓			✓	✓
Satisfaction with technology use				✓	✓					✓		✓		
Promotion of social participation				✓			✓				✓	✓	✓	
Knowledge and skill enhancement				✓							✓	✓	✓	✓
Theme 3: trust and responsibility														
Source of trust														
Health care providers		✓							✓			✓	✓	✓
Authoritative platform									✓	✓	✓	✓		
Responsibility attribution														
Individual							✓		✓				✓	
Family	✓				✓					✓			✓	
Medical personnel				✓		✓	✓		✓				✓	✓
Community			✓	✓		✓					✓		✓	
Imbalance between trust and responsibility														
Conflict between technological dependence and autonomy							✓		✓	✓				
Difficulty in verifying the authenticity of information			✓		✓	✓			✓		✓		✓	
Theme 4: adapting to digital technology														
Promoting factors														
Social support network	✓	✓	✓	✓	✓				✓	✓		✓	✓	✓
Physician-patient trust and professional resources		✓		✓					✓			✓	✓	
Technological affinity		✓	✓		✓			✓	✓	✓	✓	✓		
Crisis-driven adaptive needs				✓		✓						✓	✓	
Barrier factors														
Insufficient digital health literacy	✓		✓		✓	✓				✓	✓	✓		
Technological anxiety and negative attitudes	✓				✓	✓		✓		✓				
Usage costs	✓		✓		✓			✓						
Usability challenges related to aging	✓			✓								✓		
Generational gaps and age discrimination	✓				✓					✓				
Privacy and information security concerns					✓	✓					✓			
Technological iteration and upgrades				✓	✓					✓				

### Core Objectives

#### Obtain or Share Health Information

The core objectives of older adults using digital technologies to access and share health information center on disease management, knowledge acquisition, and access to resources. Research indicates that integrated health care applications [35], social media [39], and online communities [43] have become essential tools, enabling older adults to complete medical appointments, check test results, and obtain health information on a single platform. During the COVID-19 pandemic, the need to track vaccine effectiveness highlighted the value of technology [40]. However, the digital divide limits accessibility for certain people. As one older participant explained, *“*We need to use telemedicine services independently which is uncomfortable for us seniors. … It’s hard to apply any new digital service or product without the network approach” [42]. Similarly, older adults demonstrated active information-seeking behaviors by using specific keywords to search for diagnoses and symptoms and by comparing multiple sources to verify credibility [41]. Social media platforms also served as important channels for health information exchange within peer networks [41]. Participants valued the credibility of information sources, distinguishing expert-reviewed content from generic online information [38]. Participants also actively expanded their sources of health information through diverse channels, including public websites, television health programs, and short videos [37].

#### Maintain Health

The core objectives of health maintenance and self-management for older adults through digital technology encompass chronic disease management, health behavior incentives, and physiological indicator tracking. Patients with diabetes adjust medication regimens via online forums, such as *“*to increase one medication slightly and reduce the other one.” [43] Participants in telemedicine programs enhance self-management capabilities through training, enabling them *“*to be able to go and see your medication... add an appointment when you need your doctor, and then send your doctors the message” [40]. Some participants used digital platforms not only for exercise but also for searching health-related information such as cooking recipes, turning digital literacy into tangible health outcomes [37,41].

#### Learn Technology to Avoid Falling Behind

Some older adults proactively learn digital technologies to reduce their disconnect from societal progress, driven by both functional needs and a desire for social belonging. For some seniors, age-friendly design lowers technological barriers, *“*I’ve searched online for information about the efficacy and functions of various things” [39]. Participants in US telemedicine programs candidly acknowledge technological necessity, *“*I’m gonna be stuck... if I don’t get to learn how*”* [40], reflecting passive adaptation pressures amid widespread technological adoption. Hong Kong seniors describe persistent challenges from rapid technological iteration, *“*Sometimes I feel behind with technology because it changes so frequently*”* [42]. Some participants gained new technological skills through their involvement in health communication programs; for instance, one participant who had never used videoconferencing reported, *“*I never used Zoom before, but now I use it quite often and I use it now with my disability group.” [36]

### Behavioral Outcomes

#### Access to Medical Resources and Services

Older adults have enhanced the efficiency and accessibility of health care services by participating in community digital health communication. Using applications such as HealthHub, seniors can schedule medical appointments, view test results, and manage their medication information in one place [35,42,43]. Telemedicine platforms further streamline the diagnostic and treatment process. For instance, during the COVID-19 pandemic, medication delivery services prompted participants to remark, *“*The consultation, medication, and delivery were very reasonable, and it’s cheaper than going to a GP” [40]. Across multiple contexts, older adults similarly reported that digital communication channels reduce physical barriers to health care access, with the convenience of remote consultations being a frequently cited benefit [36,41].

#### Improve Health Status

Older adults improved both physical and mental health through participation in community digital health communication. Telemedicine training programs equipped participants with health monitoring skills and enhanced their capacity for ongoing self-care [40]. Patients with diabetes optimized disease management using technological tools, adjusting medication regimens through guidance obtained from online forums [43]. Participants also applied health information obtained through digital platforms to achieve measurable health improvements, *“*Doctor asked me to exercise. I learned an exercise from YouTube, and I followed it before my medical checkup…it helped to lower my cholesterol” [41], illustrating how digital health information can translate into tangible health outcomes when older adults possess adequate information navigation skills. Furthermore, older adults adopted traditional health practices such as moxibustion and Baduanjin exercises after engaging in community health education [37]. In a Canadian telehealth coaching program, participants valued the incremental approach to behavior change, reframing setbacks as “a new starting point, rather than…a failure” [36].

#### Promote Self-Management

Older adults achieve autonomy and personalization in health management through participation in digital technology and community health communication. Patient portals empower patients to manage their medical affairs directly [40]; patients with chronic diseases develop medication plans that are integrated with their daily routines [43]. Some seniors enhance their self-efficacy through technological learning, “You know you are kind of forced into it, and then once you feel confident, we enjoy using it all the time” [44]. Finnish seniors emphasize personal health responsibility, *“*Prevention is the patient’s responsibility for him/herself” [46]. Notably, participants reported transitioning from relying on family for health information to independently accessing and applying it, “After the doctor’s guidance, I learnt about medication by myself and no longer relied on my children” [37]. The incremental approach to behavior change was also valued, with participants appreciating the opportunity to make small, manageable changes rather than attempting comprehensive lifestyle overhauls [36].

#### Satisfaction With Technology Use

Mastering digital technology during health communication activities brings older adults a sense of accomplishment and control over their lives. After telemedicine training, participants proudly stated “My friends’ parents and grandparents don’t know as much as I do” [40]. Even those initially resistant to technology shifted their attitudes after adaptation, *“*Once you’re forced to engage and build confidence, you start enjoying it*”* [45]. The portability of tablets was particularly valued, *“*I can use it in the living room, bedroom, or anywhere*”* [44]. Participants also expressed high satisfaction with both the content and the delivery format of digital health programs, recognizing their potential community-wide benefits [38].

#### Promotion of Social Participation

Older adults experience reduced social isolation when participating in community digital health outreach activities. During the COVID-19 pandemic, telemedicine served as a vital means of social connection: *“*Well-being confined in the house, you get sick and tired of the television set... And here I can be in touch with friends” [40]. Patients with chronic diseases built support networks through online forums: *“*I know I am not alone … this experience by itself is very helpful” [43]. Social media platforms facilitated ongoing peer connections and health information exchange [41]. Digital health communication also catalyzed broader social reconnection, with participants reporting increased willingness to engage with others beyond the digital context [38]. Community health communication activities further fostered social participation through multiple peer roles, including peer pairing, community volunteering, and group leadership, with participants reporting significantly expanded social circles [37].

#### Knowledge and Skill Enhancement

Participation in community digital health communication also contributed to older adults’ acquisition of health-related knowledge and information skills. By engaging with digital health content, participants developed competencies in navigating online health information, including keyword-based searching and source verification, *“*Google is fast but no real… We look for Mayo. Mayo is quite informative” [41]. Participants also learned to critically evaluate the credibility of health information sources, distinguishing between advertisements and evidence-based content [37]. Digital health communication equipped participants with the skills necessary for independent health care access [36,40]. Engagement with expert-reviewed health content enabled participants to distinguish reliable information from unverified online sources [38], an information literacy outcome that extends beyond the specific program context.

### Trust and Responsibility

#### Source of Trust

Older adults’ trust in digital health communication was primarily built upon 2 pillars: health care providers and authoritative platforms. Regarding health care providers, their professional advice served as a core source of trust across multiple contexts. Participants emphasized the importance of established physician-patient relationships [46] and relied on physician recommendations to navigate health information sources [35]. Trust in both health coaches and health care organizations facilitated active participation in community health communication activities [36,37]. One participant noted: *“*I don’t look at him as a student. I look at him like someone who’s there to help me…he has a lot of knowledge” [36]. Regarding authoritative platforms, government-endorsed digital platforms further strengthened older adults’ trust and willingness to engage [41]. Participants stressed that eHealth platforms managed by trusted providers can deliver reliable, tailored information [46]. The perceived credibility of content also mattered: participants who encountered expert-reviewed material valued it more highly than generic online information [38,44].

#### Responsibility Attribution

Responsibility in the use of digital health technologies involves multistakeholder collaboration, encompassing a 4-dimensional interaction among individuals, families, medical personnel, and communities. Personal responsibility emphasizes proactive health management, with patients with chronic diseases independently adjusting their medication regimens using digital tools [43], while also underscoring the individual obligation to prevent diseases [46]. Family responsibilities manifest as technical guidance and financial support, with children assisting in device operation [44,47], though this sometimes undermines the autonomy of older adults. Health care professionals’ responsibilities center on professional authority, guiding technology adoption through trusted platform recommendations [46] and remote training [40]. Health coaches further facilitate health communication by providing accountability through regular follow-ups [36]. Patients with chronic diseases should optimize management strategies by integrating physicians’ advice with online information [43]. Community responsibility fosters collaborative networks through the provision of resources (eg, gamified health initiatives [39]) and infrastructure support, but resource disparities may lead to the collapse of accountability systems [42]. Community health communication activities also foster shared responsibility through peer pairing and group leadership roles [37].

#### Imbalance Between Trust and Responsibility

##### Conflict Between Technological Dependence and Autonomy

Older adults face a conflict between technological dependence and autonomy when participating in community digital health communication. Patients with diabetes gain efficient support through technological tools, but overreliance on recommendations may undermine personalized decision-making [43]. For instance, when children operate devices on their behalf, *“*My children will have to work the device” [44], it leads to stagnation in technological proficiency. European research further reveals the double-edged nature of authority dependence: older adults strictly adhere to technology platforms recommended by their family physicians [46].

##### Difficulty in Verifying the Authenticity of Information

Older adults often face challenges in verifying the authenticity of health information, primarily due to information overload, ambiguous sources, and privacy risks. When obtaining health information through social media, older adults become confused due to the mixed nature of the content [39]. Some seniors noted that conflicting online health advice intensifies the difficulty of filtering information [46]. Insufficient transparency in technological design further hinders the verification of information’s authenticity [42]. Failure to validate information may lead to a loss of trust, with some participants reverting to traditional channels due to the inability to confirm the information’s reliability [45]. However, some participants developed critical information appraisal skills, learning to distinguish between advertisements and evidence-based content [37]. Participants acknowledged the risks inherent in digital information environments, describing digital tools as having both benefits and dangers, and expressing concerns about privacy and online scams [41].

### Adapting to Digital Technology

#### Promoting Factors

##### Social Support Network

The social support network for older adults adapting to digital technologies spans 3 levels: family, peers, and community. Family support centers on intergenerational technology guidance, such as assisting with translating health information [47] or teaching device operation [45], with some older adults overcoming tech anxiety through familial encouragement [44,46]. Peer and social networks enhance the credibility of health information through shared lived experiences [35,39], and online brain health programs further extend peer support by encouraging participants to share content with family and friends [38]. Community support builds digitally inclusive ecosystems for older adults through systematic interventions, such as training programs to enhance telemedicine capabilities [40] and motivate healthy behaviors [39]. Regular follow-up sessions within health communication programs create accountability structures that motivate sustained engagement [36]. Community-based peer pairing and group activities also provide structured support for health communication participation [37].

##### Physician-Patient Trust and Professional Resources

Authoritative recommendations from health care providers are a core source of trust in health communication technologies. Older adults place greater trust in platforms endorsed by physicians, *“*You would prefer to go to your own GP who knows you already since so many years” [46]. Health care institutions simplify processes through integrated services [35] while professional resource support further reduces barriers, *“*I feel more confident now... I’m glad to have the tablet” [40]. Expert-reviewed health content was perceived as more credible than generic online sources [38], and trust in health care organizations facilitated active participation in community health communication activities [37].

##### Technological Affinity

Technological affinity manifests as older adults’ perceptions of the ease of use, learning adaptability, and emotional acceptance of digital tools. Age-friendly design enhances user comfort by simplifying interface logic and focusing on functionality [35,39]. Some technology-reluctant seniors shift their attitudes due to user-friendly interfaces; for instance, telemedicine platforms gain “gradual acceptance and reliance” through intuitive operational workflows [48]. Additionally, family guidance, gamified incentives, and authoritative guidance reduce learning resistance [44,46]. Furthermore, technological utility and controllability drive shifts in emotional attitudes, such as when older adults transition from forced usage to recognizing *“*it’s one of the daily conveniences now,” ultimately forming a dependency [45]. Active social media users demonstrated higher digital literacy and more confident engagement with digital health information [41]. Enjoyment of program design elements, such as graphics, narration, and font readability, also contributed to sustained engagement with health content [38].

##### Crisis-Driven Adaptive Needs

Older adults accessed health care services through telemedicine platforms during the pandemic. One older adult explained, *“*Right now, having not seen my doctor at her office for 6 months... I’d like to be able to have more contact with my doctor” [40]. Some participants even applied technology to track vaccine efficacy: “I will definitely continue to use the internet to monitor the efficacy of my particular vaccine and its efficacy against various variants that are developing” [40]. This pattern of adoption under crisis conditions appears largely reactive [42]. Beyond the pandemic, personal health crises also drove engagement with digital health communication. Participants who had experienced significant health events, such as cardiac stenting or thyroid surgery, placed greater emphasis on seeking and managing health information through digital tools [37]. Family history of disease, such as having a parent with dementia, motivated engagement with digital health information platforms [38].

### Barrier Factors

#### Insufficient Digital Health Literacy

Older adults commonly face challenges related to insufficient digital health literacy, specifically manifested as weak technical operational skills and difficulty in filtering health information. Some participants expressed confusion due to their inability to distinguish between credible and noncredible online health information [47]. The rapid pace of technological iteration exceeds their capacity to learn and adapt [42]. Furthermore, health knowledge gaps manifest as barriers to understanding specialized terminology. For instance, one participant noted, *“*Sometimes I still don’t understand and have to see my family doctor for explanations*”* [45]. Even among participants with adequate digital literacy, the gap between knowledge and actual use of digital health communication tools remained significant [41]. Language complexity and medical terminology in health information also posed barriers for those with lower education levels [38].

#### Technological Anxiety and Negative Attitudes

The complexity and rapid iteration of digital health technologies have led to significant technological anxiety and negative attitudes among some older adults. Research indicates that high barriers to learning these technologies are a primary contributing factor [47]. Frustration during technical operations reinforces negative attitudes, diminishing the willingness to use these tools [42]. Furthermore, technical failures trigger self-doubt, leading some older adults to abandon digital tools altogether [44]. Notably, some participants firmly maintain that face-to-face communication with physicians carries greater credibility, *“*I would still prefer in person” [48].

#### Usage Costs

Both explicit and implicit economic costs constrain the adoption of digital health technologies. Explicit costs include device purchases and subscription fees [39,45]. Implicit costs manifest as additional investments required for adapting to new technology. For instance, older adults may need to purchase translation tools or rely on their families for assistance because of language barriers [47] or frequently replace hardware due to compatibility issues [45]. Meanwhile, telepresence robots in senior living facilities face high initial acquisition costs, prompting comments such as “If I could buy Maxine at a reasonable price... I would be up for that” [48].

#### Usability Challenges Related to Aging

Age-related physiological decline directly limits older adults’ ability to operate digital health technologies. Visual impairment poses a significant barrier to using small-screen devices [47]. Deteriorating motor skills and cognitive load, particularly when distractions are present, pose significant challenges [40]. Additionally, fatigue or pain from chronic conditions can shorten the adequate usage time*,“*Our eyesight is getting worse. Devices like smartphones are even smaller and extremely difficult to view” [40]. Beyond physiological factors, accessibility challenges also include language complexity and educational disparities that hinder engagement with digital health information: *“*I live in a rural community with many people here who have not a very extensive education, the language would detract from their willingness or wanting to be part of the program” [38].

#### Generational Gaps and Age Discrimination

The absence of intergenerational support and entrenched societal biases significantly hinders the effective adoption of digital health technologies among older adults. Within family settings, technology instruction often fails due to intergenerational communication breakdowns [44], leading to diminished motivation and autonomy among older adults [47]. At the societal level, ageism manifests as a systemic underestimation of older adults’ technological capabilities [45]. Complex interface logic, overly small fonts, or a lack of voice support in some applications reflect developers’ disregard for the needs of the older population [44].

#### Privacy and Information Security Concerns

Privacy leakages and data security risks pose significant barriers to the adoption of digital health technologies among older adults. Participants commonly expressed concerns about unauthorized use of health information [45] and the potential exposure of sensitive health issues through reliance on online systems [42]. Insufficient transparency in some technological designs exacerbates trust issues. For instance, applications often fail to clearly outline data storage rules or sharing scopes, leaving older adults unclear about *“*how health data is processed and who has access to it*”* [45]. Some participants reported direct experiences with account hacking, which led to reduced use of digital communication platforms [41]. Furthermore, the potential consequences of technical failures trigger deeper fears, *“*We also have concerns about our privacy if we totally rely on the system” [42].

#### Technological Iteration and Upgrades

Rapid technological iteration and device compatibility issues create structural barriers, intensifying the alienation of older adults from digital health technologies. Frequent software upgrades lead to redundant features and altered operational logic, *“*If it is too new, we cannot catch up” [40], while pressure from outdated devices forces some participants to replace their hardware [45]. The “efficiency-first” logic of technological development overlooks the adaptation capacity of older adults. For some, frequent updates erode confidence and ultimately lead to abandonment, highlighting the imbalance between the pace of technological iteration and users’ cognitive rhythms [44].

## Discussion

### Summary of Principal Findings

This qualitative systematic review and meta-ethnography synthesized qualitative evidence on the experiences of older adults engaging with community-based digital health communication. By integrating 14 studies conducted across 8 countries and involving community-dwelling older adults aged 50 years and older with diverse health conditions and levels of digital technology experience, 4 overarching themes were identified: core objectives, behavioral outcomes, trust and responsibility, and adapting to digital technology. The synthesis was characterized predominantly by reciprocal translations, suggesting substantial commonality in older adults’ experiences across cultural and geographic contexts; no fundamentally refutational findings were identified. Nevertheless, inherent tensions emerged within shared phenomena, most notably in the dual nature of family assistance and authority-based trust.

### Objectives and Outcomes of Participating in Community Digital Health Communication

Our synthesis demonstrates that, within community-based digital health communication, older people are transitioning from recipients of medical instructions to proactive health managers [49]. This shift from passive treatment to active intervention has expanded health improvement beyond clinical indicators to include behavioral empowerment, highlighting the internalization of responsibility and awareness [50-52]. The findings are consistent with previous research on health communication [53,54].

In particular, the findings of our study indicate that digital technologies appear to have substantially reshaped the patterns of health information dissemination within communities. They have also prompted some older people to learn new technologies to avoid falling behind, reflecting both technological needs and the demand for social participation [55]. These outcomes illustrate that digital health communication supports networked interactions within communities, expanding the communication subjects from a unidirectional, provider-to-patient model to a multidirectional exchange [56]. Older people are not only information recipients but also become nodes for disseminating health information [57-59]. In this process, the older adult population builds networks of experiential empathy, fostering social connections and interactions that effectively alleviate feelings of loneliness [60].

### The Dynamic Evolution of Trust and the Transfer of Responsibility

Our study reveals that digital health technologies have profoundly reshaped the trust structures and responsibility allocation among older adults. Older participants consistently exhibit authority dependence, placing high trust in technology platforms recommended by health care professionals while valuing credibility endorsements from official institutions [61-64]. Regarding responsibility allocation, older people perceive their health responsibilities as shared among themselves, their families, health care providers, and the community [65]. We interpret this ideal multistakeholder collaboration as a potential closed-loop support system; in practice, however, this ideal is rarely fully realized [52]. Indeed, the introduction of digital media has led to notable vulnerabilities in responsibility allocation [66]. Some studies indicate that reliance on family technical assistance and medical authority can inhibit the autonomy of older people [43,44].

The synthesis also identified inherent complexities within the trust and responsibility dynamic. Our findings suggest that older adults face a paradoxical situation: the same digital environment that broadens their access to health information simultaneously overwhelms their capacity to evaluate its credibility [67]. This is consistent with previous research indicating that information overload and ambiguous sourcing pose particular challenges for populations with limited digital health literacy [68]. When older adults lack the skills to independently verify information, they may either withdraw from digital engagement or uncritically accept nonauthoritative content, both ultimately compromising health decision-making [67]. Privacy concerns further compound this complexity. Some older adults avoid data sharing due to fears of unauthorized use of health information, while insufficient transparency in technological design exacerbates their distrust of digital platforms [66]. Meanwhile, while family members’ technical assistance addresses immediate operational needs, it may simultaneously create a dependency that displaces opportunities for independent skill acquisition [69]. Digital media extends trust to intangible technological entities and shifts responsibility allocation from a person-to-person contract to a triangular relationship involving people, technology, and institutions [70-72]. This restructuring requires the community to move beyond its foundational technical support role and instead assume the critical function of coordinating responsibility allocation [73].

### Promoting and Barrier Factors of Older Adults’ Adaptation to Digital Technologies

The findings of this study indicate that older adults possess relatively abundant experience and insights regarding the application of digital technologies when participating in community-based digital health communication. The MATOA (Model for the Adoption of Technology by Older Adults) suggests that older adults’ willingness to use technology is influenced by multiple factors, including physiological aging limitations, anxiety, prerequisite knowledge, intrinsic motivation, and perceived usefulness, with education, age, and gender serving as moderating variables [74]. This study validates the applicability of MATOA in the field of health communication.

While technological affinity enhancements mitigate age-related operational barriers, the resulting privacy risks diminish the perceived benefits of digital health technologies for older adults [75]. This suggests that usability improvements alone are insufficient; they must be accompanied by transparent data governance to sustain older adults’ willingness to engage [66]. Frequent technological updates and iterations create barriers to practical applications for older adults, requiring them to enhance their digital technology skills and digital health literacy continually [76]. If relevant knowledge is not adequately disseminated, it may substantially undermine older adults’ user experience and willingness to adopt technology, deepening their negative attitudes toward technology and amplifying their anxiety about it [7,75].

Social support, as a key component of intrinsic motivation and perceived usefulness, positively drives technology adoption through community mutual aid networks and family assistance [69]. However, as discussed above, this positive role is not unconditional. Generational divides and ageism create unique barriers: the transfer of technological authority within family settings distorts the original intent of social support, while societal assumptions about capability exacerbate older adults’ technological exclusion [77].

Public health emergencies propel digital technologies from being “optional” to becoming “essential necessities,” markedly amplifying the imperative for technological adaptation beyond the intrinsic motivation inherent in the original framework of MATOA [19]. Additionally, when trust between physician and patients reaches a certain level, older people may perceive the adoption of digital technologies, such as those recommended by physicians through digital health platforms, as shifting from merely “potentially beneficial” to a “necessary safeguard” [78]. This transformative effect is not fully reflected in MATOA’s “usage expectations.” Therefore, future research may consider extending the existing model to accommodate the demands of complex scenarios.

### Implications for Practice

Based on the findings of this review, several implications for different professional groups can be drawn. For clinicians and health care providers, measures should balance technological assistance with older people’s autonomy, avoiding excessive dependence that leads to skill stagnation and insufficient support that causes digital exclusion. Systematic approaches must be implemented to enhance older people’s digital health literacy, strengthening their ability to verify health information. For community program designers and policymakers, attention must be paid to the dynamic shifts in health needs among older people, encouraging their transition from basic health management to personalized health care. The social nature of health communication within community settings should also be considered, with the design of tiered social support programs prioritizing isolated seniors, those of advanced age, and childless older individuals. For methodological and theoretical researchers, future research may consider extending the MATOA framework to accommodate the demands of complex scenarios, particularly those involving crisis-driven adoption and trust-mediated technology acceptance.

### Limitations

This study has certain limitations. Most included studies (n=12) were assessed as having moderate methodological quality, with only 2 rated as high quality. Future qualitative research should strengthen its methodological rigor. Findings from studies with lower methodological quality scores should be interpreted with caution. During the screening phase, only studies with abstracts published in Chinese or English were included, which may have resulted in the exclusion of relevant studies published in other languages, thereby limiting the representativeness of the studies and the generalizability of the findings. Additionally, our search strategy was iteratively refined through internal team discussions but was not formally peer-reviewed using the PRESS checklist, which may have introduced search-strategy bias.

### Conclusions

Drawing on 14 studies across 8 countries, this qualitative meta-ethnography takes community-based digital health communication itself as the unit of synthesis, departing from earlier reviews that have centered on the determinants of technology adoption or on clinical-pathway effectiveness. This review reframes the community as more than a setting in which digital health communication occurs; the community is itself a factor that shapes whether and how such communication works for older adults. Older adults’ engagement is best understood as an ongoing negotiation of motivation, trust, and responsibility, with that responsibility distributed unevenly across older adults, their families, peers, and health systems. These insights argue for a shift in practice and policy: policymakers, community program designers, and clinicians should move beyond efficiency-oriented service delivery toward building collective community capacity, tiered support for the most isolated older adults, and protection of older adults’ autonomy and capacity to verify health information.

## Supplementary material

10.2196/89179 Multimedia Appendix 1 Search terms and outcomes across databases.

10.2196/89179 Multimedia Appendix 2 Representative examples of the development of third-order constructs through the meta-ethnographic synthesis (n=14).

10.2196/89179 Multimedia Appendix 3 Characteristics of included studies (n=14).

10.2196/89179 Checklist 1 PRISMA checklist.

10.2196/89179 Checklist 2 PRISMA 2020 expanded checklist.

10.2196/89179 Checklist 3 PRISMA 2020 for abstracts checklist.

10.2196/89179 Checklist 4 PRISMA-S checklist.

10.2196/89179 Checklist 5 ENTREQ checklist.

10.2196/89179 Checklist 6 SWiM checklist.

10.2196/89179 Multimedia Appendix 4 The Critical Appraisal Skills Programme quality assessment of included studies (n=14).

## Abbreviations

CASP: Critical Appraisal Skills Programme
eMERGe: Meta-Ethnography Reporting Guidance
ENTREQ: Enhancing Transparency in Reporting the Synthesis of Qualitative Research
GRADE-CERQual: Grading of Recommendations Assessment, Development, and Evaluation—Confidence in the Evidence from Reviews of Qualitative Research
MATOA: Model for the Adoption of Technology by Older Adults
PRESS: Peer Review of Electronic Search Strategies
PRISMA: Preferred Reporting Items for Systematic Reviews and Meta-Analyses
PRISMA-S: PRISMA Extension for Reporting Literature Searches in Systematic Reviews
PROSPERO: International Prospective Register of Systematic Reviews
SAGE: Study on Global Ageing and Adult Health
SWiM: Synthesis Without Meta-Analysis
WHO: World Health Organization
