# Supplementary material for: Digital Health Communication Engagement Experiences of Older Adults in Community Contexts: Qualitative Systematic Review and Meta-Ethnography
Source: J Med Internet Res. 2026 Aug 5;28:e89179. doi: 10.2196/89179 (PMC13441116; doi:10.2196/89179)
Supplement: Supplementary file 4 [file jmir-v28-e89179-s007.pdf]

PRISMA-S Checklist

Section/topic	#	Checklist item	Location(s) Reported
**INFORMATION SOURCES AND METHODS**			
Database name	1	Name each individual database searched, stating the platform for each.	P5, Methods – Information sources
Multi-database searching	2	If databases were searched simultaneously on a single platform, state the name of the platform, listing all of the databases searched.	P5, Methods – Information sources; Search strategy
Study registries	3	List any study registries searched.	P5, Methods – Information sources
Online resources and browsing	4	Describe any online or print source purposefully searched or browsed (e.g., tables of contents, print conference proceedings, web sites), and how this was done.	P5, Methods – Information sources
Citation searching	5	Indicate whether cited references or citing references were examined, and describe any methods used for locating cited/citing references (e.g., browsing reference lists, using a citation index, setting up email alerts for references citing included studies).	P5, Methods – Information sources
Contacts	6	Indicate whether additional studies or data were sought by contacting authors, experts, manufacturers, or others.	P5, Methods – Information sources
Other methods	7	Describe any additional information sources or search methods used.	P5, Methods –

			Information sources
**SEARCH STRATEGIES**			
Full search strategies	8	Include the search strategies for each database and information source, copied and pasted exactly as run.	P5, Methods – Search strategy; Multimedia Appendix 1
Limits and restrictions	9	Specify that no limits were used, or describe any limits or restrictions applied to a search (e.g., date or time period, language, study design) and provide justification for their use.	P5, Methods – Search strategy
Search filters	10	Indicate whether published search filters were used (as originally designed or modified), and if so, cite the filter(s) used.	P5, Methods – Search strategy
Prior work	11	Indicate when search strategies from other literature reviews were adapted or reused for a substantive part or all of the search, citing the previous review(s).	P5, Methods – Search strategy
Updates	12	Report the methods used to update the search(es) (e.g., rerunning searches, email alerts).	P5, Methods – Search strategy
Dates of searches	13	For each search strategy, provide the date when the last search occurred.	Multimedia Appendix 1
**PEER REVIEW**			
Peer review	14	Describe any search peer review process.	P5, Methods – Search strategy
**MANAGING RECORDS**			
Total Records	15	Document the total number of records identified from each database and other information sources.	P8-9, Results – Study selection; Figure 1 (PRISMA

			flow diagram)
Deduplication	16	Describe the processes and any software used to deduplicate records from multiple database searches and other information sources.	P 5, Methods – Selection process; P 9, Figure 1

PRISMA-S: An Extension to the PRISMA Statement for Reporting Literature Searches in Systematic Reviews
Rethlefsen ML, Kirtley S, Waffenschmidt S, Ayala AP, Moher D, Page MJ, Koffel JB, PRISMA-S Group.
Last updated February 27, 2020.
