# Supplementary material for: Digital Health Communication Engagement Experiences of Older Adults in Community Contexts: Qualitative Systematic Review and Meta-Ethnography
Source: J Med Internet Res. 2026 Aug 5;28:e89179. doi: 10.2196/89179 (PMC13441116; doi:10.2196/89179)
Supplement: Supplementary file 7 [file jmir-v28-e89179-s001.docx]

**Multimedia Appendix 1: Search terms and outcomes across databases**

| **Database: PubMed**  **Search time: 2025.03.24; 2026.04.30; 2026.06.09** | | |
| --- | --- | --- |
| **Search** | **Query** | **Items found** |
| #1 | "aged"[MeSH Terms] OR "geriatric*"[Title/Abstract] OR "retire*"[Title/Abstract] OR "older people"[Title/Abstract] OR "older adults"[Title/Abstract] OR "elder people"[Title/Abstract] OR "elder adults"[Title/Abstract] OR "aged people"[Title/Abstract] OR "aged adults"[Title/Abstract] OR "retired people"[Title/Abstract] OR "retired adults"[Title/Abstract] OR "senior"[Title/Abstract] OR "baby boomer"[Title/Abstract] OR "centenarians"[Title/Abstract] | 4,037,427 |
| #2 | "community"[Title/Abstract] OR "neighbourhood"[Title/Abstract] OR "neighborhood"[Title/Abstract] OR "resident* area"[Title/Abstract] OR "resident* district"[Title/Abstract] | 833,680 |
| #3 | "digital"[Title/Abstract] OR "ehealth"[Title/Abstract] OR "e-health"[Title/Abstract] OR "online"[Title/Abstract] OR "electronic"[Title/Abstract] OR "mhealth"[Title/Abstract] OR "ICT"[Title/Abstract] OR "digital health"[MeSH Terms] OR "telemedicine"[MeSH Major Topic] | 1,096,180 |
| #4 | "health"[Title/Abstract] OR "medical"[Title/Abstract] OR "well-being"[Title/Abstract] OR "fitness"[Title/Abstract] | 4,846,419 |
| #5 | "knowledge"[Title/Abstract] OR "fact"[Title/Abstract] OR "advice"[Title/Abstract] OR "information"[Title/Abstract] OR "guidance"[Title/Abstract] | 3,305,058 |
| #6 | #4 AND #5 | 932,203 |
| #7 | "popular*"[Title/Abstract] OR "disseminat*"[Title/Abstract] OR "teach*"[Title/Abstract] OR "educ*"[Title/Abstract] OR "public participation in science"[Title/Abstract] OR "knowledge transfer"[Title/Abstract] | 1,515,861 |
| #8 | #6 AND #7 | 234,865 |
| #9 | "health communication"[MeSH Terms] | 3,887 |
| #10 | #8 OR #9 | 238,147 |
| #11 | "qualitative research"[MeSH Terms] OR "qualitative method"[Title/Abstract] OR "qualitative study"[Title/Abstract] OR "qualitative research"[Title/Abstract] OR "phenomenolog*"[Title/Abstract] OR "lived experience*"[Title/Abstract] OR "hermeneutic*"[Title/Abstract] OR "Heideggerian"[Title/Abstract] OR "husserl*"[Title/Abstract] OR "grounded theory"[Title/Abstract] OR "ethnograph*"[Title/Abstract] OR "case study"[Title/Abstract] OR "action research"[Title/Abstract] OR "discourse analy*"[Title/Abstract] OR "interview*"[Title/Abstract] OR "focus group*"[Title/Abstract] OR "participant observ*"[Title/Abstract] OR "field note*"[Title/Abstract] OR "content analy*"[Title/Abstract] OR "thematic analy*"[Title/Abstract] OR "Colaizzi"[Title/Abstract] OR "Giorgi"[Title/Abstract] OR "Van manen"[Title/Abstract] OR "constant comparison"[Title/Abstract] OR "constant comparative analysis"[Title/Abstract] | 847,497 |
| #12 | #1 AND #2 AND #3 AND #10 AND #11 | 268 |

| **Database: EMBASE**  **Search time: 2025.03.24; 2026.04.30; 2026.06.09** | | |
| --- | --- | --- |
| **Search** | **Query** | **Items found** |
| #1 | 'geriatric*':ab,ti OR 'retire*':ab,ti OR 'older people':ab,ti OR 'older adults':ab,ti OR 'elder people':ab,ti OR 'elder adults':ab,ti OR 'aged people':ab,ti OR 'aged adults':ab,ti OR 'retired people':ab,ti OR 'retired adults':ab,ti OR 'senior':ab,ti OR 'baby boomer':ab,ti OR 'centenarians':ab,ti | 478,098 |
| #2 | 'community':ab,ti OR 'neighbourhood':ab,ti OR 'neighborhood':ab,ti OR 'resident* area':ab,ti OR 'resident* district':ab,ti | 1,042,872 |
| #3 | 'digital':ab,ti OR 'ehealth':ab,ti OR 'e-health':ab,ti OR 'online':ab,ti OR 'electronic':ab,ti OR 'tele*':ab,ti OR 'mhealth':ab,ti OR 'ICT':ab,ti | 1,620,691 |
| #4 | 'health':ab,ti OR 'medical':ab,ti OR 'well-being':ab,ti OR 'fitness':ab,ti | 6,496,346 |
| #5 | 'knowledge':ab,ti OR 'fact':ab,ti OR 'advice*':ab,ti OR 'information':ab,ti OR 'guidance':ab,ti | 4,315,020 |
| #6 | #4 AND #5 | 1,298,708 |
| #7 | 'popular*':ab,ti OR 'disseminat*':ab,ti OR 'teach*':ab,ti OR 'educ*':ab,ti OR 'public participation in science':ab,ti OR 'knowledge transfer':ab,ti | 1,989,437 |
| #8 | #6 AND #7 | 326,910 |
| #9 | 'health communication':ab,ti | 5510 |
| #10 | #8 OR #9 | 331,091 |
| #11 | 'qualitative research':ab,ti OR 'qualitative method':ab,ti OR 'qualitative study':ab,ti OR phenomenology*:ab,ti OR 'lived experience*':ab,ti OR hermeneutic*:ab,ti OR heideggerian:ab,ti OR husserl*:ab,ti OR 'grounded theory':ab,ti OR ethnograph*:ab,ti OR 'case study':ab,ti OR 'action research':ab,ti OR 'discourse analy*':ab,ti OR interview*:ab,ti OR 'focus group*':ab,ti OR 'participant observer*':ab,ti OR 'field note*':ab,ti OR 'content analy*':ab,ti OR 'thematic analy*':ab,ti OR colaizzi:ab,ti OR giorgi:ab,ti OR 'van manen':ab,ti OR 'constant comparison':ab,ti OR 'constant comparative analysis':ab,ti | 1,038,200 |
| #12 | #1 AND #2 AND #3 AND #10 AND #11 | 349 |

| **Database: CINAHL Complete**  **Search time: 2025.03.24; 2026.04.30; 2026.06.09** | | |
| --- | --- | --- |
| **Search** | **Query** | **Items found** |
| S1 | TI (geriatric* OR retire* OR older people OR older adults OR elder people OR elder adults OR aged people OR aged adults OR retired people OR retired adults OR senior OR baby boomer OR centenarians) OR SU (geriatric* OR retire* OR older people OR older adults OR elder people OR elder adults OR aged people OR aged adults OR retired people OR retired adults OR senior OR baby boomer OR centenarians) OR AB (geriatric* OR retire* OR older people OR older adults OR elder people OR elder adults OR aged people OR aged adults OR retired people OR retired adults OR senior OR baby boomer OR centenarians) | 249,996 |
| S2 | TI (community OR neighbourhood OR neighborhood OR "resident* area" OR "resident* district") OR SU (community OR neighbourhood OR neighborhood OR "resident* area" OR "resident* district") OR AB (community OR neighbourhood OR neighborhood OR "resident* area" OR "resident* district") | 414,603 |
| S3 | TI (digital OR ehealth OR e-health OR online OR electronic OR tele* OR mhealth OR ICT) OR SU (digital OR ehealth OR e-health OR online OR electronic OR tele* OR mhealth OR ICT) OR AB (digital OR ehealth OR e-health OR online OR electronic OR tele* OR mhealth OR ICT) | 448,881 |
| S4 | TI (health OR medical OR well-being OR fitness) OR SU (health OR medical OR well-being OR fitness) OR AB (health OR medical OR well-being OR fitness) | 2,846,521 |
| S5 | TI (knowledge OR fact OR advice OR information OR guidance) OR SU (knowledge OR fact OR advice OR information OR guidance) OR AB (knowledge OR fact OR advice OR information OR guidance) | 1,009,019 |
| S6 | S4 AND S5 | 538,233 |
| S7 | TI (popular* OR disseminat* OR teach* OR educ* OR "public participation in science" OR "knowledge transfer") OR SU (popular* OR disseminat* OR teach* OR educ* OR "public participation in science" OR "knowledge transfer") OR AB (popular* OR disseminat* OR teach* OR educ* OR "public participation in science" OR "knowledge transfer") | 1,055,221 |
| S8 | S6 AND S7 | 178,901 |
| S9 | TI ("health communication") OR SU ("health communication") OR AB ("health communication") | 2,683 |
| S10 | S8 OR S9 | 180,936 |
| S11 | TI(qualitative research OR qualitative method OR qualitative study OR qualitative research OR phenomenolog* OR lived experience* OR hermeneutic* OR Heideggerian OR husserl* OR grounded theory OR ethnograph* OR case study OR action research OR discourse analy* OR interview* OR focus group* OR participant observ* OR field note* OR content analy* OR thematic analy* OR Colaizzi OR Giorgi OR Van manen OR constant comparison OR constant comparative analysis) OR SU (qualitative research OR qualitative method OR qualitative study OR qualitative research OR phenomenolog* OR lived experience* OR hermeneutic* OR Heideggerian OR husserl* OR grounded theory OR ethnograph* OR case study OR action research OR discourse analy* OR interview* OR focus group* OR participant observ* OR field note* OR content analy* OR thematic analy* OR Colaizzi OR Giorgi OR Van manen OR constant comparison OR constant comparative analysis) OR AB (qualitative research OR qualitative method OR qualitative study OR qualitative research OR phenomenolog* OR lived experience* OR hermeneutic* OR Heideggerian OR husserl* OR grounded theory OR ethnograph* OR case study OR action research OR discourse analy* OR interview* OR focus group* OR participant observ* OR field note* OR content analy* OR thematic analy* OR Colaizzi OR Giorgi OR Van manen OR constant comparison OR constant comparative analysis) | 784,080 |
| S12 | S1 AND S2 AND S3 AND S10 AND S11 | 214 |

| **Database: Web of Science Core Collection**  **Search time: 2025.03.24; 2026.04.30; 2026.06.09** | | |
| --- | --- | --- |
| **Search** | **Query** | **Items found** |
| #1 | TS=(geriatric* OR retire* OR older people OR older adults OR elder people OR elder adults OR aged people OR aged adults OR retired people OR retired adults OR senior OR baby boomer OR centenarians) | 1,556,380 |
| #2 | TS=(community OR neighbourhood OR neighborhood OR "resident* area" OR "resident* district") | 2,333,916 |
| #3 | TS=(digital OR ehealth OR e-health OR online OR electronic OR tele* OR mhealth OR ICT) | 3,928,560 |
| #4 | TS=(health OR medical OR well-being OR fitness) | 6,274,696 |
| #5 | TS=(knowledge OR fact OR advice OR information OR guidance) | 8,073,016 |
| #6 | #4 AND #5 | 1,224,653 |
| #7 | TS=(popular* OR disseminat* OR teach* OR educ* OR "public participation in science" OR "knowledge transfer") | 3,654,781 |
| #8 | #6 AND #7 | 298,877 |
| #9 | TS=("health communication") | 11,481 |
| #10 | #8 OR #9 | 307,519 |
| #11 | TS=(qualitative research OR qualitative method OR qualitative study OR qualitative research OR phenomenolog* OR lived experience* OR hermeneutic* OR Heideggerian OR husserl* OR grounded theory OR ethnograph* OR case study OR action research OR discourse analy* OR interview* OR focus group* OR participant observ* OR field note* OR content analy* OR thematic analy* OR Colaizzi OR Giorgi OR Van manen OR constant comparison OR constant comparative analysis) | 7,842,423 |
| #12 | #1 AND #2 AND #3 AND #10 AND #11 | 932 |

| **Database: ProQuest Health & Medical Collection**  **Search time: 2025.03.24; 2026.04.30; 2026.06.09** | | |
| --- | --- | --- |
| **Search** | **Query** | **Items found** |
| S1 | ti(geriatric* OR retire* OR older people OR older adults OR elder people OR elder adults OR aged people OR aged adults OR retired people OR retired adults OR senior OR baby boomer OR centenarians) OR ab(geriatric* OR retire* OR older people OR older adults OR elder people OR elder adults OR aged people OR aged adults OR retired people OR retired adults OR senior OR baby boomer OR centenarians) OR su(geriatric* OR retire* OR older people OR older adults OR elder people OR elder adults OR aged people OR aged adults OR retired people OR retired adults OR senior OR baby boomer OR centenarians) | 787,139 |
| S2 | ti(community OR neighbourhood OR neighborhood OR "resident* area" OR "resident* district") OR ab(community OR neighbourhood OR neighborhood OR "resident* area" OR "resident* district") OR su(community OR neighbourhood OR neighborhood OR "resident* area" OR "resident* district") | 600,536 |
| S3 | ti(digital OR ehealth OR e-health OR online OR electronic OR tele* OR mhealth OR ICT) OR ab(digital OR ehealth OR e-health OR online OR electronic OR tele* OR mhealth OR ICT) OR su(digital OR ehealth OR e-health OR online OR electronic OR tele* OR mhealth OR ICT) | 692,895 |
| S4 | ti(health OR medical OR well-being OR fitness) OR ab(health OR medical OR well-being OR fitness) OR su(health OR medical OR well-being OR fitness) | 5,379,351 |
| S5 | ti(knowledge OR fact OR advice OR information OR guidance) OR ab(knowledge OR fact OR advice OR information OR guidance) OR su(knowledge OR fact OR advice OR information OR guidance) | 1,721,819 |
| S6 | [S4] AND [S5] | 779,672 |
| S7 | ti(popular* OR disseminat* OR teach* OR educ* OR public participation in science OR knowledge transfer) OR ab(popular* OR disseminat* OR teach* OR educ* OR public participation in science OR knowledge transfer) OR su(popular* OR disseminat* OR teach* OR educ* OR public participation in science OR knowledge transfer) | 1,027,882 |
| S8 | [S6] AND [S7] | 196,125 |
| S9 | ti("health communication") OR ab("health communication") OR su("health communication") | 5,332 |
| S10 | [S8] OR [S9] | 198,913 |
| S11 | ti(qualitative research OR qualitative method OR qualitative study OR qualitative research OR phenomenolog* OR lived experience* OR hermeneutic* OR Heideggerian OR husserl* OR grounded theory OR ethnograph* OR case study OR action research OR discourse analy* OR interview* OR focus group* OR participant observ* OR field note* OR content analy* OR thematic analy* OR Colaizzi OR Giorgi OR Van manen OR constant comparison OR constant comparative analysis) | 148,245 |
| S12 | [S1] AND [S2] AND [S3] AND [S10] AND [S11] | 71 |

| **Database: PsycINFO**  **Search time: 2025.03.24; 2026.04.30; 2026.06.09** | | |
| --- | --- | --- |
| **Search** | **Query** | **Items found** |
| S1 | KW ( geriatric* OR retire* OR "older people" OR "older adults" OR "elder people" OR "elder adults" OR "aged people" OR "aged adults" OR "retired people" OR "retired adults" OR senior OR "baby boomer" OR centenarians ) | 52,010 |
| S2 | TI ( community OR neighbourhood OR neighborhood OR "resident* area" OR "resident* district" ) OR AB ( community OR neighbourhood OR neighborhood OR "resident* area" OR "resident* district" ) OR KW ( community OR neighbourhood OR neighborhood OR "resident* area" OR "resident* district" ) | 412,265 |
| S3 | TI ( digital OR ehealth OR e-health OR online OR electronic OR tele* OR mhealth OR ICT ) OR AB ( digital OR ehealth OR e-health OR online OR electronic OR tele* OR mhealth OR ICT ) OR KW ( digital OR ehealth OR e-health OR online OR electronic OR tele* OR mhealth OR ICT ) | 324,152 |
| S4 | TI ( health OR medical OR well-being OR fitness ) OR AB ( health OR medical OR well-being OR fitness ) OR KW ( health OR medical OR well-being OR fitness ) | 1,101,589 |
| S5 | TI ( knowledge OR fact OR advice OR information OR guidance ) OR AB ( knowledge OR fact OR advice OR information OR guidance ) OR KW ( knowledge OR fact OR advice OR information OR guidance ) | 1,057,427 |
| S6 | S4 AND S5 | 242,378 |
| S7 | TI ( popular* OR disseminat* OR teach* OR educ* OR "public participation in science" OR "knowledge transfer" ) OR AB ( popular* OR disseminat* OR teach* OR educ* OR "public participation in science" OR "knowledge transfer" ) OR KW ( popular* OR disseminat* OR teach* OR educ* OR "public participation in science" OR "knowledge transfer" ) | 963,252 |
| S8 | S6 AND S7 | 72,118 |
| S9 | TI ( "health communication" ) OR AB ( "health communication" ) OR KW ( "health communication" ) | 4,458 |
| S10 | S8 OR S9 | 75,755 |
| S11 | TI ( "qualitative research" OR "qualitative method" OR "qualitative study" OR phenomenolog* OR "lived experience*" OR hermeneutic* OR Heideggerian OR husserl* OR "grounded theory" OR ethnograph* OR "case study" OR "action research" OR "discourse analy*" OR interview* OR "focus group*" OR "participant observ*" OR "field note*" OR "content analy*" OR "thematic analy*" OR Colaizzi OR Giorgi OR "Van Manen" OR "constant comparison" OR "constant comparative analysis" ) OR AB ( "qualitative research" OR "qualitative method" OR "qualitative study" OR phenomenolog* OR "lived experience*" OR hermeneutic* OR Heideggerian OR husserl* OR "grounded theory" OR ethnograph* OR "case study" OR "action research" OR "discourse analy*" OR interview* OR "focus group*" OR "participant observ*" OR "field note*" OR "content analy*" OR "thematic analy*" OR Colaizzi OR Giorgi OR "Van Manen" OR "constant comparison" OR "constant comparative analysis" ) OR KW ( "qualitative research" OR "qualitative method" OR "qualitative study" OR phenomenolog* OR "lived experience*" OR hermeneutic* OR Heideggerian OR husserl* OR "grounded theory" OR ethnograph* OR "case study" OR "action research" OR "discourse analy*" OR interview* OR "focus group*" OR "participant observ*" OR "field note*" OR "content analy*" OR "thematic analy*" OR Colaizzi OR Giorgi OR "Van Manen" OR "constant comparison" OR "constant comparative analysis" ) | 686,331 |
| S12 | S1 AND S2 AND S3 AND S10 AND S11 | 16 |

| **Database: Scopus**  **Search time: 2025.03.24; 2026.04.30; 2026.06.09** | | |
| --- | --- | --- |
| **Search** | **Query** | **Items found** |
| S1 | TITLE-ABS-KEY ( geriatric* OR retire* OR "older people" OR "older adults" OR "elder people" OR "elder adults" OR "aged people" OR "aged adults" OR "retired people" OR "retired adults" OR senior OR "baby boomer" OR centenarians ) | 697,253 |
| S2 | TITLE-ABS-KEY ( community OR neighbourhood OR neighborhood OR "resident* area" OR "resident* district" ) | 2,940,060 |
| S3 | TITLE-ABS-KEY ( digital OR ehealth OR e-health OR online OR electronic OR tele* OR mhealth OR ICT ) | 6,394,775 |
| S4 | TITLE-ABS-KEY ( health OR medical OR well-being OR fitness ) | 11,127,939 |
| S5 | TITLE-ABS-KEY ( knowledge OR fact OR advice OR information OR guidance ) | 12,082,200 |
| S6 | S4 AND S5 | 2,234,642 |
| S7 | TITLE-ABS-KEY ( popular* OR disseminat* OR teach* OR educ* OR "public participation in science" OR "knowledge transfer" ) | 5,640,401 |
| S8 | S6 AND S7 | 591,838 |
| S9 | TITLE-ABS-KEY ( "health communication" ) | 16,801 |
| S10 | S8 OR S9 | 603,540 |
| S11 | TITLE-ABS-KEY ( "qualitative research" OR "qualitative method" OR "qualitative study" OR phenomenolog* OR "lived experience*" OR hermeneutic* OR Heideggerian OR husserl* OR "grounded theory" OR ethnograph* OR "case study" OR "action research" OR "discourse analy*" OR interview* OR "focus group*" OR "participant observ*" OR "field note*" OR "content analy*" OR "thematic analy*" OR Colaizzi OR Giorgi OR "Van Manen" OR "constant comparison" OR "constant comparative analysis" ) | 3,498,794 |
| S12 | S1 AND S2 AND S3 AND S10 AND S11 | 407 |
| All | (TITLE-ABS-KEY ( geriatric* OR retire* OR "older people" OR "older adults" OR "elder people" OR "elder adults" OR "aged people" OR "aged adults" OR "retired people" OR "retired adults" OR senior OR "baby boomer" OR centenarians )) AND (TITLE-ABS-KEY ( community OR neighbourhood OR neighborhood OR "resident* area" OR "resident* district" )) AND (TITLE-ABS-KEY ( digital OR ehealth OR e-health OR online OR electronic OR tele* OR mhealth OR ICT )) AND (( TITLE-ABS-KEY ( "health communication" ) ) OR ( ( TITLE-ABS-KEY ( popular* OR disseminat* OR teach* OR educ* OR "public participation in science" OR "knowledge transfer" ) ) AND ( ( TITLE-ABS-KEY ( knowledge OR fact OR advice OR information OR guidance ) ) AND ( TITLE-ABS-KEY ( health OR medical OR well-being OR fitness ) ) ) )) AND (TITLE-ABS-KEY ( "qualitative research" OR "qualitative method" OR "qualitative study" OR phenomenolog* OR "lived experience*" OR hermeneutic* OR Heideggerian OR husserl* OR "grounded theory" OR ethnograph* OR "case study" OR "action research" OR "discourse analy*" OR interview* OR "focus group*" OR "participant observ*" OR "field note*" OR "content analy*" OR "thematic analy*" OR Colaizzi OR Giorgi OR "Van Manen" OR "constant comparison" OR "constant comparative analysis" )) | 407 |

| **Database: CNKI**  **Search time: 2025.03.24; 2026.04.30; 2026.06.09** | |
| --- | --- |
| **Search Query** | **Items found** |
| (TKA=('老年'+'老龄'+'退休')) AND (TKA=('社区'+'街道')) AND (TKA=('数字'+'电子'+'在线'+'互联网'+'新媒体'+'智慧'+'数智')) AND ((TKA=('健康'+'医学'+'保健') AND TKA=('科普'+'普及'+'传播'+'宣传')) OR TKA='健康传播') AND (TKA=('定性研究'+'定性方法'+'质性研究'+'现象学'+'生活经验'+'解释学'+'海德格尔'+'胡塞尔'+'扎根理论'+'民族志'+'案例研究'+'行动研究'+'话语分析'+'访谈'+'焦点小组'+'参与者观察'+'田野笔记'+'内容分析'+'主题分析'+'比较分析')) | 5 |

| **Database: WANFANG**  **Search time: 2025.03.24; 2026.04.30; 2026.06.09** | |
| --- | --- |
| **Search Query** | **Items found** |
| ((主题:("老年" OR "老龄" OR "退休")) AND (主题:("社区" OR "街道")) AND (主题:("数字" OR "电子" OR "在线" OR "互联网" OR "新媒体" OR "智慧" OR "数智")) AND (((主题:("健康" OR "医学" OR "保健") AND 主题:("科普" OR "普及" OR "传播" OR "宣传")) OR 主题:"健康传播")) AND (主题:("定性研究" OR "定性方法" OR "质性研究" OR "现象学" OR "生活经验" OR "解释学" OR "海德格尔" OR "胡塞尔" OR "扎根理论" OR "民族志" OR "案例研究" OR "行动研究" OR "话语分析" OR "访谈" OR "焦点小组" OR "参与者观察" OR "田野笔记" OR "内容分析" OR "主题分析" OR "比较分析"))) | 20 |
