# Supplementary material for: Digital Health Communication Engagement Experiences of Older Adults in Community Contexts: Qualitative Systematic Review and Meta-Ethnography
Source: J Med Internet Res. 2026 Aug 5;28:e89179. doi: 10.2196/89179 (PMC13441116; doi:10.2196/89179)
Supplement: Supplementary file 8 [file jmir-v28-e89179-s002.docx]

**Multimedia Appendix 2. Representative Examples of Development of Third-Order Constructs Through Meta-Ethnographic Synthesis (N=14).**

| Study | First-Order Constructs (Participant Quotations) | Second-Order Constructs (Original Authors’ Interpretations) | Third-Order Constructs: Themes | Third-Order Constructs: Sub-themes |
| --- | --- | --- | --- | --- |
| Zhang YC et al. (2023), Singapore | *“I use the HealthHub app…I found it very useful. And it does have family programs and health tips that I can go to find out more.”*  *“Google and read the review of the medicine, then go to some forum. I will check as many sources as I can.”* | Older adults used ICTs and social networks for health information seeking, health maintenance, responding to symptoms, and telemedicine access. | Theme 1: Core Objectives | Obtain/Share Health Information |
| Neumann AV et al. (2023), America | *“I will definitely continue to use the internet to monitor the efficacy of my particular vaccine and its efficacy against various variants that are developing.”* | Participants accessed healthcare services, health information, and gained a sense of purpose and control through digital technology use. | Theme 1: Core Objectives | Maintain Health |
| Neumann AV et al. (2023), America | *“I’d like to learn how to be able to use the internet ‘cause everything is switching over to that, and I’m gonna be stuck…if I don’t get to learn how.”* | Participants were motivated by the need to adapt to a technology-dependent world and avoid social exclusion. | Theme 1: Core Objectives | Learn Technology to Avoid Falling Behind |
| Zhang YC et al. (2023), Singapore | *“I tried to do a virtual or teleconsult when I self-tested COVID…After the consultation, they sent the medication in within 3 to 4 hours. The consultation, medication, and delivery were very reasonable.”* | Technology facilitated access to healthcare services including teleconsultation, medication delivery, and appointment management. | Theme 2: Behavioral Outcomes | Access to Medical Resources and Services |
| Kyaw MY et al. (2026), Singapore | *“Doctor asked me to exercise. I learnt an exercise from YouTube, and I followed it before my medical checkup…it helped to lower my cholesterol.”* | eHealth literacy skills directly linked to health behaviors; digital technology use behavior transformed to visible health outcomes. | Theme 2: Behavioral Outcomes | Improved Health Status / Promotion of Self-Management |
| Wang XX et al. (2025), China | *“After the doctor’s guidance, I learnt about medication by myself and no longer relied on my children.”*  *“After leading the group to do health exercises, I feel that I still have a role to play.”* | Participants transitioned from passive reception to active engagement in health self-management; social roles expanded through peer pairing, volunteering, and group leadership. | Theme 2: Behavioral Outcomes | Promotion of Self-Management / Promotion of Social Participation |
| Akenine U et al. (2020), Europe | *“You would prefer to go to your own GP who knows you already since so many years, rather than commit to someone you do not know.”* | Trust in the healthcare provider was a prerequisite to engage in prevention programmes; participants valued long-standing relationships and professional expertise. | Theme 3: Trust and Responsibility | Source of Trust: Healthcare Providers |
| Kyaw MY et al. (2026), Singapore | *“Very secure, it was apps from government, our government is very good.”* | Government endorsement made technology feel safe; participants felt secure using government-developed apps and platforms. | Theme 3: Trust and Responsibility | Source of Trust: Authoritative Platform |
| Zibrik L et al. (2015), Canada | *“We tried to learn from our children but they do not have patience to spend more time to teach us.”*  *“My children will have to work the device.”* | Reliance on family technical assistance could inhibit autonomy; intergenerational communication breakdowns led to diminished motivation. | Theme 3: Trust and Responsibility | Imbalance: Conflict Between Technological Dependence and Autonomy |
| Glowacki EM et al. (2021), America | *“Whenever I get a new piece of technology, I always hand it off to my kids, so they can kind of navigate me through it.”* | Family support, peer learning, and community resources served as key facilitators for technology adoption and comfort. | Theme 4: Adapting to Digital Technology | Promoting Factor: Social Support Network |
| Arthanat S et al. (2024), America | *“I was a little nervous at first, and then I became very comfortable with it.”* | Residents’ comfort with and understanding of the robot increased with more exposure; convenience of access facilitated adoption. | Theme 4: Adapting to Digital Technology | Promoting Factor: Technological Affinity |
| Zibrik L et al. (2015), Canada | *“As seniors, we don’t know about those things. I don’t know how to use an iPad. The best one for me is those old Microsoft Windows computers. If it is too new, we cannot catch up.”* | Insufficient digital health literacy was a major barrier; older adults lacked skills and experience with newer digital devices and interfaces. | Theme 4: Adapting to Digital Technology | Barrier Factor: Insufficient Digital Health Literacy |

***Note:*** *This table presents representative examples from each theme to illustrate the reciprocal translation process. First-order constructs are direct participant quotations (presented in italics). Second-order constructs are the original study authors’ interpretations. Third-order constructs are the synthesized themes and sub-themes generated through the current meta-ethnography.*
