# Supplementary material for: Digital Health Communication Engagement Experiences of Older Adults in Community Contexts: Qualitative Systematic Review and Meta-Ethnography
Source: J Med Internet Res. 2026 Aug 5;28:e89179. doi: 10.2196/89179 (PMC13441116; doi:10.2196/89179)
Supplement: Supplementary file 9 [file jmir-v28-e89179-s003.docx]

**Multimedia Appendix 3. Characteristics of Included Studies (N=14).**

| Author (Year), Country | Aim(s) | Study Design | Qualitative Sample & Demographics | Data Collection Method | Analysis | Key Qualitative Findings |
| --- | --- | --- | --- | --- | --- | --- |
| Zibrik L et al. (2015), Canada | To explore barriers to and facilitators of e-health for chronic disease self-management among immigrant Chinese and Punjabi older adults in British Columbia. | Mixed methods (QUAN + qual) | Qualitative component: n=55 (28 Punjabi + 27 Chinese older adults)  Age: Not specified (older adults)  Sex: Not specified  Setting: Community; iCON public health events  Health status: Chronic disease patients  Sampling: Event-based recruitment (at iCON public health events) | Focus group discussions exploring e-health barriers for chronic disease self-management | Thematic analysis | Barriers: Language barriers; health literacy; lack of accessibility; negative attitudes toward technology; perceived negative health impact; physical limitations; intergenerational barriers  Facilitators: Family support; social inclusion; community-based health management programs |
| Zhang YC et al. (2023), Singapore | To explore the lived experiences of older adults regarding health information-seeking behaviors and related technology use. | Qualitative (phenomenological) | Sampling size: n=15  Age: 55-80 years (mean 65.8, SD 6)  Sex: 9 female, 6 male  Setting: Community-dwelling; various housing types in Singapore  Ethnicity: Chinese, Malay, Indian  Sampling: Convenience + snowball | Semi-structured interviews; 13 online (video-recorded) + 2 face-to-face; mean duration 39 minutes | Thematic analysis | Purposes: Health information seeking; health maintenance; teleconsultation; tele-education  Technology considerations: Perceived usefulness; comprehensiveness of features; ease of use; performance/quality; social network recommendations; cost; rewards; credibility |
| Pan TH et al. (2024), Korea | To measure digital skill levels among community-dwelling older adults; determine internet use characteristics; investigate associations between digital skills and health outcomes; explore how Korean older adults use digital technologies for healthy ageing. | Mixed methods (explanatory sequential) | Qualitative component: n=26  Age: 65+ years  Sex: Not specified  Setting: Community-based health promotion activity participants  Sampling: Convenience | Focus group discussions; sessions lasted 20-60 minutes; explored how and why older adults use digital technology for healthy ageing | Thematic analysis | Conditions: Hardware availability; age-friendly adaptations (voice assistants); digital health literacy; supportive environment  Purposes: Social connection; accessing health information; participation in health promotion activities |
| Neumann AV et al. (2023), America | To test the effectiveness of the "Tech Allies for Health" telemedicine training program during COVID-19, and to understand participants' goals and experiences. | Mixed methods (QUAN + qual) | Qualitative component: n=19  Age: 65+ years  Sex: Not specified  Setting: Community-academic partnership  Sampling: Convenience | Semi-structured interviews; pre-intervention (motivations, technology/healthcare access experiences) and post-intervention (program impact, feedback) | Thematic analysis | Motivations: Accessing healthcare/treatment; accessing health information; desire to learn internet use  Outcomes: Accessing healthcare services; learning digital health information access; sense of accomplishment; enhanced social participation; promoted psychological well-being |
| Glowacki EM et al. (2021), America | To explore experiences of middle-aged and older adults using ICTs and Health IT; to identify facilitators and barriers of Health IT interventions. | Qualitative | Sampling size: n=41  Age: 41-86 years  Sex: 26 female, 15 male  Race: 32 White, 7 African American, 2 Hispanic  Setting: Community-dwelling  Sampling: Purposive (part of longitudinal community ethnography) | Semi-structured interviews about experiences using Health IT | Inductive analysis | Cognition: Use willingness (resistance, forced adoption, growing preference)  Conditions: Digital health literacy; comfort level  Access: Infrastructure; health information security  Support: Family support; peer learning; intergenerational gaps; ageism |
| Cheng L et al. (2023), Hong Kong, China | To identify challenges and barriers of telemedicine for smart ageing in Hong Kong communities; to explore recommendations for mental health and telemedicine adoption; to consider future directions post-pandemic. | Mixed methods（explanatory and descriptive) | Sampling size: n=57  Age: 60-70+ years  Sex: Not specified  Setting: Community; SMART program participants  Sampling: Purposive | 5 focus group discussions facilitated by a leader and 2-3 project assistants; sessions 1.5-2 hours | Content analysis | Motivations: Loneliness; lack of support; desire for well-being; health awareness; stress/disease management; health knowledge acquisitionBarriers: HCPs not proactively disseminating information; peer reluctance; digital skills gaps; privacy concerns; organizational barriers; insufficient policy support |
| Bernhard G et al. (2017), Germany | To describe challenges perceived by T2DM patients in self-managing their medication regimen; to investigate strategies and the role of support networks. | Qualitative | Sampling size: n=25  Age: 64 ± 8.6, 49-77  Sex: 7 female, 18 male  Chronic conditions: Mean 3 comorbidities; complex medication regimens (≥5 medicines/day)  Setting: Community-dwelling  Sampling: Purposive | 4 focus group discussions (6-8 participants each); sessions 110-130 minutes; audio/video recorded; recruitment until thematic saturation | Content analysis (SFMF framework) | Roles/Outcomes: Focusing on illness/medication needs; developing personalized routines; cultivating medication awareness; enhancing treatment confidence; activating healthcare, community, family/friend support; exchanging experiences in online forums |
| Arthanat S et al. (2024), America | To identify facilitators and barriers to implementing a telepresence robot (Maxine) health program in senior living facilities. | Qualitative | Sampling size: n=10  Age: 65 years and older  Sex: Not specified  Setting: Community-dwelling  Sampling: Purposive | Semi-structured interviews; explored perceptions of Maxine, feasibility/usability for telemedicine delivery (UTAUT framework) | Content analysis | Facilitators: Increased comfort/understanding with exposure; perceived long-term value  Barriers: Persistent preference for in-person sessions; initial cost concerns |
| Akenine U et al. (2020), Europe (Finland, France, Netherlands) | To explore attitudes, facilitators, and barriers to engaging with an e-health self-management prevention program among older adults at increased risk for CVD and dementia. | Qualitative | Sampling size: n=56  Age: 65+ years  Sex:25 female,31 male  Health risk: Increased CVD/dementia risk  Setting: Community-dwelling; HATICE project participants across 3 countries  Sampling: Purposive | 3 rounds of focus groups (total 8 sessions) across Finland, France, and Netherlands; topics covered prevention, internet use, HCP relationships, platform layout, motivation | Grounded theory | Attitudes: Trust in primary care providers; belief in patient responsibility  Purposes: Accessing reliable health information; implementing information into practice  Facilitators: Trust in HCPs/platforms; good HCP-patient relationship  Barriers: Inability to distinguish credible information; dementia stigma |
| Saravanan A et al. (2024), America | To understand first-hand experiences and challenges of older adults using smart tablets post-COVID-19; to gather suggestions for age-appropriate training materials for accessing reliable online health information. | Qualitative (focused pilot intervention) | Sampling size: n=13  Age: Older adults (65+ years)  Sex: 11 female, 2 male  Setting: Two retirement homes in Illinois  Sampling: Purposive | 3 in-person focus groups (two groups of 4, one of 5); conducted after training sessions on smart tablet use | Thematic analysis | Attitudes: Portability valued; technology anxiety; excitement/enthusiasm; technology use as learning process; desire for self-sufficiency  Facilitators: Social support (family, peers)Barriers: Lack of experience; insufficient age-friendly design; difficulty evaluating information quality; misleading ads; anxiety from rapid updates |
| Kyaw MY et al. (2026), Singapore | To investigate eHealth literacy (eHL) and its determinants among community-dwelling older adults in Singapore; to examine the role of eHL in digital health technology usage; to explore how older adults use digital technology for health information and service access. | Mixed methods (explanatory sequential, QUAN > qual) | Qualitative component: n=23  Age: 65-80 years  Sex: 12 male, 11 female  Ethnicity: Predominantly Chinese  Setting: Active ageing centres in urban districts of Singapore (Tampines, Ang Mo Kio, Bendemeer, Clementi, Whampoa)  Sampling: Purposive (Digital technology users selected for FGDs) | 5 focus group discussions; 3 in English, 1 in Cantonese, 1 mixed; each 60-90 minutes; audio-recorded and transcribed verbatim; guided by piloted discussion guides with open-ended questions; eHL theory and TAM as theoretical scaffold | Deductive thematic analysis (eHL + TAM framework) | Theme 1 - Navigation competency: Keyword search; source verification (Mayo Clinic as trusted source; cross-source comparison)  Theme 2 - Social media as bridge: WhatsApp/Facebook for information exchange; active ageing centre groups  Theme 3 - Apps for healthy ageing: Motivation through rewards (Healthy 365); health maintenance via YouTube/Zoom; government endorsement; privacy/scam concerns |
| Philip G et al. (2025), Canada | To explore the usability and perceived benefits of Brain Health PRO (BHPro), a 45-week online educational program designed to promote healthy lifestyle behaviors and reduce dementia risk, over a 12-week pilot period. | Mixed methods (pilot study; qualitative component reported) | Sampling size:n=8  Age: 68-83 years  Sex: 4 male, 4 female  Education: 12-18 years  Location: British Columbia (3), New Brunswick (3), Ontario (2)  Setting: Rural (5), Urban (3)  Cognitive status: Cognitively intact, SCI, or MCI; at increased dementia risk  Sampling: Purposive | 2 virtual focus groups at 6 weeks and 12 weeks; each 2 hours duration; explored accessibility, likability, feedback, perceived benefits, barriers, personalization; transcribed verbatim; field notes documented | Thematic analysis | Content: Likability/accessibility; informative (expert-reviewed, not 'Google-type' information); language barriers for lower literacy  Mechanics: Positive design (graphics, narration, font); technical glitches resolved with support; digital notebook less useful; personalized risk profile valued  Engagement: Increased motivation; behavioral changes (diet, social network, sleep); desire for continuous feedback; family sharing of knowledge |
| Wang XX et al. (2025), China | To develop and examine the impact of a community healthy self-help education model for older adults (CHSE-O) on healthy behavior and active health awareness; to explore how social participation and peer support foster motivation and behavioral change. | Mixed methods (12-month pre-post self-controlled trial + qual) | Qualitative component:n=11  Age:69-79  Sex: 8 female, 3 male  Setting: 5 communities in Shanghai, China  Sampling: Convenience (from intervention participants) | Semi-structured interviews after 12-month intervention; focused on willingness to manage health, behavioral changes, competence enhancement, attitude changes, comparisons of education models, suggestions; each 20-40 minutes; audio-recorded; transcribed verbatim; saturation confirmed after 11th interview | Thematic analysis | Willingness: Health crises as driver; trust in healthcare organizations; reducing family burdenBehavioral changes: Adjusted diet/exercise; diversified health information sources; ability to distinguish ads from science  Social role change: From passive receiver to active contributor (volunteer, group leader, peer pairing)Education model comparison: Medical staff-led: authoritative, systematic; Peer-organized: flexible, interactive, life-relevant  Positive aging: Self-efficacy improvement; shift from 'cure' to 'prevention' |
| Yang MC et al. (2026), Canada | To qualitatively describe older adult (≥65 years) experiences participating in a student-delivered Community Outreach TeleheAlth program for COVID education and Health promotion (COACH). | Qualitative descriptive | Sampling size:n=24  Age: Mean 73.1 (male), 74.1 (female)  Sex: 10 male, 14 female  Race: 83.3% Caucasian  Chronic diseases: 75% had ≥2 chronic conditions  Location: Urban (46%), Rural/suburban (54%)  Education: Majority bachelor's degree or higher  Marital status: 54% married/common law  Setting: Community-dwelling across British Columbia  Sampling: Stratified purposive | One-on-one semi-structured interviews via Zoom; 30-45 minutes; conducted 2 weeks after completing COACH program; audio-recorded; field notes taken; interview guide refined throughout data collection; recruited until data sufficiency | Codebook thematic analysis | Knowledge - Gaining new: Exercise videos; online social groups; behavior change strategies (goal setting, action planning, incremental change); learning Zoom  Knowledge - Reinforcing old: Validating existing chronic disease management knowledge; refreshing previous health education  Coach as motivational support: Accountability through follow-up; coach as catalyst; student coaches valued for knowledge and drive; mutual learning benefit |

***Note:*** *For mixed-methods studies, only the qualitative component sample characteristics are reported. CASP = Critical Appraisal Skills Programme qualitative research checklist (maximum score 20). CVD = cardiovascular disease; FGD = focus group discussion; HCP = healthcare provider; ICT = information and communication technology; MCI = mild cognitive impairment; SCI = subjective cognitive impairment; SFMF = Self- and Family Management Framework; T2DM = type 2 diabetes mellitus; TAM = Technology Acceptance Model; UTAUT = Unified Theory of Acceptance and Use of Technology.*
