# Supplementary material for: Digital Health Communication Engagement Experiences of Older Adults in Community Contexts: Qualitative Systematic Review and Meta-Ethnography
Source: J Med Internet Res. 2026 Aug 5;28:e89179. doi: 10.2196/89179 (PMC13441116; doi:10.2196/89179)
Supplement: Supplementary file 10 [file jmir-v28-e89179-s010.docx]

**Multimedia Appendix 4.** CASP quality assessment of included studies (N=14)

| Items | Zibrik L et Al. (2015), Canada | Zhang Y C et Al. (2023), Singapore | Pan T H et Al. (2024), Korea | Neumann A V et Al. (2023), America | Glowacki E M et Al. (2021), America | Cheng L et Al. (2023), Hongkong, China | Bernhard G et Al. (2017), Germany | Arthanat S et Al. (2024), America | Akenine U et Al. (2020), Europe (Finland, France and Netherlands) | Saravanan A et Al. (2024), America | Kyaw M Y et al. (2026), Singapore | Philip G et al. (2025), Canada | Wang X X et al. (2025), China | Yang M C et al. (2026), Canada |
| --- | --- | --- | --- | --- | --- | --- | --- | --- | --- | --- | --- | --- | --- | --- |
| 1. Was there a clear statement of the aims of  the research？ | + | + | + | +/- | + | + | + | + | + | + | + | + | + | + |
| 2. Is a qualitative methodology appropriate？ | + | + | + | + | + | + | + | + | + | + | + | + | + | + |
| 3. Was the research design appropriate to  address the aims of the research？ | +/- | + | + | + | + | + | + | + | + | + | + | + | + | + |
| 4. Was the recruitment strategy appropriate to  the aims of the research？ | +/- | + | + | + | + | + | + | + | + | + | +/- | + | + | + |
| 5. Was the data collected in a way that  addressed the research issue？ | + | + | + | + | + | + | + | + | + | + | + | + | + | + |
| 6. Has the relationship between researcher and  participants been adequately considered? | - | - | - | - | + | - | + | - | - | +/- | +/- | +/- | - | +/- |
| 7. Have ethical issues been taken into  consideration？ | +/- | + | + | +/- | + | + | + | + | +/- | +/- | + | + | + | + |
| 8. Was the data analysis sufficiently rigorous？ | + | + | + | + | + | +/- | + | + | + | + | + | +/- | + | + |
| 9. Is there a clear statement of findings  ？ | + | + | + | + | + | + | + | + | + | + | + | + | + | + |
| 10. How valuable is the research？ | + | + | + | + | + | + | + | + | + | + | + | + | + | + |
| CASP Total Score | 15 | 18 | 18 | 16 | 20 | 17 | 20 | 18 | 17 | 18 | 18 | 18 | 18 | 18 |

Note: “+” = Yes (2 points); “+/−” = Can't tell (1 point); “−” = No (0 points)
